# Posterior-lateral basal forebrain cholinergic nuclei hypoconnectivity in the inferior frontal cortex is associated with verbal fluency deficits in schizophrenia

**DOI:** 10.1038/s41537-026-00824-0

**Published:** 2026-11-11

**Authors:** Sofia Eickhoff, Alexandra Korda, Christina Andreou, Stefan Borgwardt, Mihai Avram

**Affiliations:** Department of Psychiatry and Psychotherapy, University of Luebeck, Luebeck, Germany

**Keywords:** Psychosis, Schizophrenia

## Abstract

Cognitive impairment is a core feature of schizophrenia and remains insufficiently addressed by current treatments. Increasing evidence implicates the cholinergic system, particularly the basal forebrain cholinergic nuclei (BFCN), in cognitive dysfunction. While structural alterations of the BFCN have been reported, alterations in their intrinsic functional connectivity (iFC) remain unclear. The present study examined the iFC of anterior and posterior BFCN subdivisions (aBFCN, pBFCN) in schizophrenia and tested associations with cognitive performance. Resting-state functional magnetic resonance imaging data from the COBRE dataset were analyzed, including 73 healthy controls (HC) and 51 patients with schizophrenia (SCZ). Seed-based connectivity analyses were performed for aBFCN and pBFCN. Group differences were assessed voxel-wise, and group-dependent associations with phonemic verbal fluency (FAS) and processing speed (BACS-SC) were tested using hierarchical regression controlling for age, sex, IQ, and smoking status; verbal learning and memory (HVLT) was examined as a specificity analysis. Exploratory molecular mapping used normative PET-derived neurotransmitter maps, supplemented by spatial-autocorrelation-preserving null models. Distinct connectivity patterns of aBFCN and pBFCN were observed, consistent with previous reports in HC. Compared with HC, SCZ showed reduced pBFCN connectivity with a cluster in the right inferior frontal gyrus (pars opercularis; BA44; *p* = 0.018, FWE-corrected), whereas no significant differences were observed for aBFCN. The Group × pBFCN–IFG connectivity interaction was significant for FAS (*p* = 0.014) but not BACS-SC (*p* = 0.762) or HVLT (*p* = 0.514). In SCZ, higher pBFCN–IFG connectivity was associated with better FAS performance. Descriptive molecular mapping showed above-average representation of M1 and α4β2 receptor maps as well as several non-cholinergic targets; however, no molecular target showed significant spatial correspondence after spatial-autocorrelation-aware testing and FDR correction. These findings provide evidence for selective pBFCN hypoconnectivity with the right inferior frontal gyrus in schizophrenia. Its association with verbal fluency suggests that disrupted pBFCN connectivity with the inferior frontal cortex may contribute to altered executive control processes involved in verbal fluency in schizophrenia. Together, these findings extend previous evidence of BFCN abnormalities in schizophrenia by demonstrating a systems-level functional alteration of pBFCN–cortical connectivity and its relationship with cognitive performance.

## Introduction

Schizophrenia (SCZ) is a chronic and heterogeneous psychiatric disorder characterized by positive, negative, and cognitive symptoms, among others^1^. Notably, cognitive impairments—including deficits in attention, memory, processing speed, and executive function—are particularly persistent, strongly predict functional outcome, and remain insufficiently treated by current pharmacological and non-pharmacological treatment approaches^2–4^. Despite their clinical relevance, the neurobiological mechanisms underlying these deficits remain incompletely understood.

Although dopaminergic and glutamatergic dysfunction have long dominated pathophysiological models of SCZ^5,6^, increasing evidence implicates the cholinergic system, particularly in relation to cognition^7,8^. The basal forebrain cholinergic nuclei (BFCN) provide the major source of cortical acetylcholine projections and innervate prefrontal, limbic, and hippocampal regions that are critical for attention, learning, and memory^9–11^. At the systems level, cholinergic signaling is thought to support attentional control and the flexible coordination of distributed cortical systems^10,12^.

Converging evidence from post-mortem and molecular imaging studies indicates cholinergic abnormalities in SCZ. These include alterations in muscarinic and nicotinic receptor systems, with particular evidence for M1/M4- and α7-related dysfunction^13–17^. Such alterations have been linked to cognitive impairment as well as positive and negative symptoms^10^. Recent meta-analytic work further supports widespread cholinergic alterations and their relevance for cognitive dysfunction in SCZ^18^. Pharmacological studies provide additional support: Anticholinergic medication burden has been consistently associated with poorer cognitive performance^19–21^, whereas pro-cholinergic interventions have demonstrated beneficial effects on cognition and psychopathology^22–25^. Notably, xanomeline–trospium has demonstrated efficacy in randomized controlled trials, with robust effects on psychotic symptoms and emerging evidence for cognitive improvement^22–25^. Together, these findings underscore the translational relevance of the cholinergic system as a candidate target for cognitive symptoms in SCZ.

Structural neuroimaging studies further implicate BFCN abnormalities in schizophrenia. Reduced basal forebrain volumes have been associated with cognitive impairments^26,27^. However, it remains unclear whether these alterations primarily reflect local structural abnormalities in the BFCN or disrupted functional interactions with distributed cortical systems^10^. Addressing this question is important, as functional connectivity may provide a systems-level readout of how neuromodulatory nuclei relate to distributed cortical systems.

Resting-state functional magnetic resonance imaging (rs-fMRI) has emerged as a powerful approach for characterizing intrinsic functional connectivity (iFC) of the brain^28,29^. In SCZ, altered iFC has been reported across distributed brain systems and linked to cognitive dysfunction^30,31^. However, despite increasing evidence for cholinergic involvement, iFC patterns of the BFCN themselves have not yet been systematically examined, despite prior work highlighting this as an important open question^10^.

The BFCN comprises anatomically and functionally distinct subregions. Although earlier models proposed multiple subdivisions^32^, more recent functional connectivity work by Fritz et al.^33^ supports a parsimonious division into anterior-medial and posterior-lateral clusters with distinct, less overlapping connectivity profiles. Importantly, the anterior BFCN (aBFCN) shows preferential functional connectivity with limbic and medial temporal regions, consistent with a role in learning and memory, whereas the posterior BFCN (pBFCN) is more strongly integrated with lateral frontoparietal and transmodal cortical networks implicated in higher-order cognitive and executive functions^10,33^. Previous structural MRI studies have furthermore demonstrated alterations involving anterior and posterior BFCN subdivisions in schizophrenia^26,27^. Together, these findings provide a rationale for examining aBFCN and pBFCN functional connectivity separately and for relating their connectivity patterns to cognitive functions corresponding to their distinct network organizations.

Previous resting-state fMRI studies using the COBRE dataset have demonstrated widespread alterations of functional brain organization in schizophrenia using whole-brain, network-level, and data-driven approaches^34–36^. These approaches provide important macro-scale characterizations of schizophrenia-related dysconnectivity but have not specifically examined the functional connectivity of the BFCN and its anterior and posterior subdivisions. In the present study, we therefore applied an established functional BFCN parcellation to the COBRE dataset to investigate subdivision-specific iFC alterations in SCZ compared with HC. The selection of phonemic verbal fluency (assessed via the F-A-S verbal fluency task (FAS)) was driven by its requirement for rapid and dynamic allocation of executive control across multiple cognitive processes^37,38^, which are modulated by subcortical cholinergic input^9–12^. The choice of FAS was also motivated by our prior work examining structural BFCN alterations across the schizophrenia spectrum, in which posterior BFCN morphology showed a trend-level association with poorer phonemic verbal fluency in patients with first-episode psychosis^27^. We therefore sought to test whether altered functional coupling of the BFCN is associated with verbal fluency performance in schizophrenia. Processing speed (assessed via the BACS Symbol Coding Task (BACS-SC)) was retained as a second primary cognitive outcome for continuity with previous structural BFCN findings^26,27^, while verbal learning and memory (assessed via the Hopkins Verbal Learning Test (HVLT)) was included based on the subdivision-specific functional organization of the aBFCN^10,33^ and was additionally examined as a specificity analysis for the observed pBFCN connectivity alteration.

Based on this subdivision-specific functional organization, we hypothesized that schizophrenia would be associated with distinct alterations in the iFC of the aBFCN and pBFCN. Specifically, we expected aBFCN connectivity alterations to preferentially involve limbic and medial temporal circuitry and to be associated with verbal learning and memory performance (HVLT). In contrast, we expected pBFCN connectivity alterations to preferentially involve lateral frontoparietal and transmodal circuitry and to be associated with cognitive performance involving executive function and processing speed, including phonemic verbal fluency (FAS) and processing speed (BACS-SC). Cognitive associations were subsequently examined for connectivity alterations showing significant between-group differences.

## Methods

### Participants

Imaging, clinical, and cognitive data for patients with SCZ and HC were obtained from the publicly available Center for Biomedical Research Excellence (COBRE) dataset^39^. The original COBRE study was approved by the Institutional Review Board of the University of New Mexico (UNM), and all participants provided written informed consent. No new data were collected for the present study. The COBRE dataset was selected primarily because it allowed us to build directly on previous structural investigations of BFCN subdivisions in the same cohort, providing multimodal continuity between structural and functional characterization of the BFCN within an established sample. COBRE further provides comprehensive clinical and cognitive phenotyping together with resting-state functional MRI and structural MRI, enabling BFCN functional connectivity to be examined in relation to clinically and cognitively relevant measures. Finally, its public availability facilitates transparency, reproducibility, and independent reanalysis.

The original dataset comprised 73 HC and 72 patients. Patients were recruited as part of the COBRE study and met DSM-IV criteria for schizophrenia or schizoaffective disorder^40^, confirmed using the Structured Clinical Interview for DSM-IV Axis I Disorders^41^, with diagnostic consensus reached by two research psychiatrists^39^. The original COBRE protocol required patients to demonstrate both retrospective and prospective clinical stability. Retrospective stability was established from psychiatric records documenting no change in symptomatology or in the type or dose of psychotropic medication during the three months preceding study referral. Prospective stability was assessed during three consecutive weekly visits and at each imaging assessment; clinical symptom ratings were obtained within one week of each neuroimaging assessment^39^. Thus, patients were clinically stable rather than acutely ill at imaging. Exclusion criteria for patients included a history of neurological disorders, head trauma resulting in loss of consciousness for more than five minutes, intellectual disability, and active substance abuse or dependence (except nicotine) within the preceding 12 months; participants were additionally required to have a negative toxicology screen for drugs of abuse at study entry^39^. HC had no personal history of Axis I disorders, no history of substance abuse, and no first-degree relatives with psychosis. In the SCZ group, 71 of 72 individuals were receiving antipsychotic medication, predominantly second-generation antipsychotics; a minority received first-generation antipsychotics or benzodiazepines.

Following preprocessing and quality control, participants with excessive head motion (mean framewise displacement (FD) > 0.3 mm) were excluded from further analysis.

### Clinical assessment

Psychopathology was assessed using the Positive and Negative Syndrome Scale (PANSS)^42^.

Additional clinical variables included age of onset, duration of illness, current antipsychotic medication dosage expressed as chlorpromazine equivalents (CPZ) based on established conversion guidelines^43^, and anticholinergic burden scores (ACB) based on established scales^44^.

### Cognitive assessment

Processing speed was assessed using the Symbol Coding Task of the Brief Assessment of Cognition in Schizophrenia (BACS-SC)^45^, which measures processing speed and attentional efficiency through rapid symbol-digit matching.

Verbal fluency was assessed using the F-A-S Phonemic Verbal Fluency Test (FAS)^46^, in which participants generated words beginning with the letters F, A, and S within 60 s per letter.

Verbal learning and memory were assessed using the Hopkins Verbal Learning Test (HVLT) total score^47^.

### Data acquisition and preprocessing

Structural and functional MRI data were obtained from the COBRE dataset^39^ and acquired on a 3 T Siemens Trio scanner using a 12-channel radiofrequency coil. Detailed MRI acquisition parameters are provided in the Supplementary Methods.

Preprocessing was performed using the Configurable Pipeline for the Analysis of Connectomes (C-PAC, version 1.7.0), following the default preconfigured pipeline unless otherwise specified. Preprocessing steps included slice-timing correction, motion correction, scrubbing based on framewise displacement using a threshold of 0.3 mm, intensity normalization, nuisance signal regression, band-pass filtering (0.01–0.1 Hz), co-registration to anatomical space, and normalization to Montreal Neurological Institute (MNI) space (3 mm isotropic resolution) using FSL FLIRT/FNIRT. Nuisance signal regression was performed using a unified multiple linear regression model including anatomical component-based noise correction (aCompCor), retaining five principal components each from white-matter and cerebrospinal-fluid signals. Head-motion effects were modeled using the Friston 24-parameter model, including six motion parameters, their temporal derivatives, and the corresponding squared terms. Global signal regression was not applied.

### Intrinsic functional connectivity of the BFCN

The iFC of the aBFCN and pBFCN was examined using whole-brain seed-to-voxel correlation analysis implemented in C-PAC. The aBFCN and pBFCN seed regions were derived from the previously published functional parcellation of the cholinergic basal forebrain by Fritz et al.^33^ and were used without modification. Briefly, Fritz et al. defined a comprehensive cholinergic basal forebrain region of interest in MNI space based on existing stereotactic atlases and clustered voxel-wise resting-state functional connectivity profiles using k-means clustering. The two-cluster solution yielded anterior-medial and posterior-lateral subdivisions, referred to here as the aBFCN and pBFCN, respectively. These functional subdivisions closely mirror the cytoarchitectonic distinction between the septal/diagonal band complex (Ch1–Ch3; aBFCN) and the nucleus basalis of Meynert (Ch4; pBFCN)^48,49^. The anatomical extent of the seed masks corresponds to the original published parcellation, and no additional parcellation or modification was performed in the present study (Supplementary Fig. 1). When represented on the 3-mm isotropic functional analysis grid, the aBFCN and pBFCN seed masks comprised 82 and 49 voxels, respectively, corresponding to spatial volumes of 2214 and 1323 mm³.

For each participant, the mean time series was extracted from each seed and correlated with all voxels within the whole-brain gray matter mask. Correlation coefficients were Fisher z-transformed to obtain subject-specific connectivity maps.

### Statistical analyses

#### Demographic, cognitive, and clinical characteristics

Group differences in demographic, clinical, and cognitive variables were assessed using two-sample t-tests for continuous variables and χ^2^ tests for categorical variables, as appropriate. Variables of interest included age, sex, smoking status, and IQ.

#### Voxel-wise statistical analysis of BFCN intrinsic functional connectivity, controlling for age and sex

Voxel-wise statistical analyses were conducted using SPM12. One-sample t-tests were used to generate group-level connectivity maps for each seed and group. Group differences between HC and patients with SCZ were assessed using two-sample t-tests assuming unequal variance. Analyses were restricted to voxels within an explicit whole-brain gray matter mask.

Age and sex were included as covariates in the voxel-wise between-group analyses to account for major demographic influences on iFC while maintaining a parsimonious whole-brain model. Additional cognitive and behavioral covariates were not included because the primary objective was to identify diagnostic group differences in BFCN connectivity. IQ was intentionally excluded from diagnostic group comparisons to avoid over-controlling for variance intrinsic to the disorder phenotype. Statistical significance was defined using a cluster-defining threshold of *p* < 0.001 (uncorrected), with clusters considered significant at *p* < 0.05 FWE-corrected at the cluster level and a minimum cluster extent of *k* ≥ 15 voxels. All statistical tests were two-sided unless otherwise specified.

#### Exploratory molecular mapping analyses

Exploratory molecular mapping was performed by mapping the schizophrenia-related pBFCN hypoconnectivity cluster onto normative PET-derived neurotransmitter atlases from the Hansen atlas collection^50^. PET receptor/transporter enrichment was quantified using z-scores relative to a cortical gray matter reference distribution to characterize the neurochemical modulatory profile of the identified connectivity cluster.

First, the significant schizophrenia-related pBFCN hypoconnectivity cluster was extracted from the group-level analysis and resliced into the space of each PET receptor/transporter map using nearest-neighbor interpolation within a Python-based analysis pipeline.

PET maps included dopaminergic, serotonergic, cholinergic, glutamatergic, GABAergic, histaminergic, noradrenergic, cannabinoid, and opioid markers derived from the Hansen PET atlas^50^.

To obtain a cortical reference distribution, a cortical mask based on the Harvard–Oxford cortical atlas (cort-maxprob-thr25-2mm) was used. The cortical mask was resampled into the space of each PET map, and only positive PET voxels within the cortical mask were included in the reference distribution.

For each PET map, the mean PET signal intensity was extracted within the schizophrenia-related pBFCN hypoconnectivity cluster. Relative enrichment scores were calculated using z-transformation relative to the cortical PET reference distribution:

$${{{\rm{z}}}}=({{{\rm{mean}}}}\_{{{\rm{cluster}}}}-{{{\rm{mean}}}}\_{{{\rm{cGM}}}})/{{{\rm{SD}}}}\_{{{\rm{cGM}}}}$$

where *mean_cluster* represents the mean PET signal within the hypoconnectivity cluster, *mean_cGM* the mean PET signal across the cortical reference mask, and *SD_cGM* the corresponding cortical standard deviation.

For receptors represented by multiple PET datasets, receptor-level mean z-scores were calculated across PET maps. Results were visualized using a receptor-level spider plot.

To determine whether molecular spatial correspondences exceeded those expected from the intrinsic spatial organization of the PET maps, we additionally performed a spatial-autocorrelation-preserving null-model analysis using BrainSMASH^51^. For each of the 19 receptor/transporter targets, a receptor-specific valid cortical PET domain was defined; for targets represented by multiple datasets, the valid domain was the intersection of valid voxels, and standardized PET maps were averaged voxel-wise. The spatial-null test was restricted to voxels located within both the pBFCN–IFG cluster and the respective valid cortical domain. BrainSMASH generated 500 spatial surrogate maps per target that approximately matched the spatial autocorrelation structure of the empirical molecular map. Empirical and surrogate maps were standardized within their respective domains, and the mean standardized value across cluster voxels was used as the test statistic. Two-sided empirical *p*-values were calculated from the surrogate distributions, with Benjamini–Hochberg FDR correction across the 19 molecular targets.

#### Associations of BFCN intrinsic functional connectivity with anticholinergic burden and smoking in schizophrenia, controlling for age and sex

To examine whether BFCN functional connectivity was associated with anticholinergic burden or smoking status, additional whole-brain multiple regression analyses were performed separately for the aBFCN and pBFCN seeds within the SCZ group. Analyses were restricted to patients with complete data for ACB, smoking status, age, and sex (*n* = 40). ACB, smoking status, age, and sex were entered simultaneously as covariates. Positive and negative associations with ACB and smoking status were assessed using separate contrasts while controlling for the remaining covariates. Consistent with the primary seed-based analyses, results were assessed using a voxel-level threshold of p < .001 uncorrected with a minimum cluster extent of *k* ≥ 15 voxels, followed by cluster-level FWE correction at *p* < 0.05. Significant ACB-associated pBFCN clusters were compared with the pBFCN–IFG group-difference cluster, and spatial overlap was quantified as the voxel-wise intersection between binarized cluster maps.

#### Association between pBFCN–IFG connectivity and cognitive performance, controlling for age, sex, IQ, and smoking

To examine cognitive associations with disorder-related connectivity alterations, mean functional connectivity values were extracted from pBFCN clusters showing a significant main effect of diagnostic group in the primary voxel-wise analysis. Statistical analyses were conducted in Jamovi (Version 2.6.44). Analyses were conducted in the subsample with available cognitive data (HC: *n* = 55; SCZ: *n* = 40). Outliers were identified separately within each diagnostic group and for each cognitive outcome using Tukey’s criterion (values below Q1 − 1.5 × IQR or above Q3 + 1.5 × IQR) and were excluded prior to the respective analyses. This resulted in the exclusion of three HC participants from FAS, one SCZ participant from BACS-SC, and two HC participants from HVLT. Regression analyses were based on complete cases for all variables included in the respective model.

Two-step hierarchical multiple linear regression analyses were performed separately for FAS and BACS-SC. As an additional specificity analysis, the same approach was applied to the HVLT total score. In step 1, group, pBFCN–IFG functional connectivity, age, sex, IQ, and smoking status were entered as predictors; in step 2, the Group × pBFCN–IFG connectivity interaction was added. Improvement in model fit was assessed using Δ*R*² and the corresponding *F*-test. To account for the two primary cognitive outcomes (FAS and BACS-SC), a Bonferroni-corrected significance threshold of α = 0.025 was applied. HVLT was considered a specificity analysis and was not included in this primary correction. The inclusion of IQ and smoking status, together with age and sex, was intended to reduce confounding of connectivity–cognition associations by general intellectual ability and smoking behavior.

#### Follow-up and sensitivity analyses

For a significant Group × connectivity interaction, group-specific simple-slope models and partial correlations were calculated controlling for age, sex, IQ, and smoking status; group-specific partial correlations were compared using Fisher’s r-to-z transformation, accounting for the number of covariates. For significant group-specific associations within SCZ, sensitivity analyses further adjusted for PANSS-negative symptom severity, ACB, and CPZ. Regression assumptions were evaluated using residual-versus-fitted and Q–Q plots, the Shapiro–Wilk test, variance inflation factors, Cook’s distance, Mahalanobis distance, and the Durbin–Watson statistic. A separate volume sensitivity analysis was performed in participants with available pBFCN volume data (HC: *n* = 56; SCZ: *n* = 41). Extracted pBFCN–IFG connectivity was the dependent variable; group, age, and sex were entered in step 1, followed by TIV-corrected pBFCN volume in step 2. Improvement in model fit was assessed using Δ*R*² and the corresponding *F*-test. Structural preprocessing and volume definition for this cohort have been described previously^26^.

## Results

### Demographic, cognitive, and clinical characteristics

Demographic, clinical, and cognitive characteristics are summarized in Table 1. Following preprocessing and quality control, 21 participants were excluded because of excessive head motion. The final sample for intrinsic functional connectivity analyses consisted of 73 HCs (age range: 18–65 years; 53 males) and 51 SCZ participants (age range: 19–57 years; 43 males).

**Table 1:** Participant demographics, clinical scores, and cognitive scores.

Variable	Group	*N*	Missing	Mean	SD	Statistic	*p*
Age (years)	HC	73	0	38.60	12.1	*t*(122) = 1.48	0.140
	SCZ	51	0	35.20	13.0		
Female (%)	HC	73	0	27.40	—	χ²(1) = 2.36	0.125
	SCZ	51	0	15.69	—		
Age of onset (years)	HC	0	73	—	—	—	—
	SCZ	40	11	20.53	8.30		
Duration of illness (years)	HC	0	73	—	—	—	—
	SCZ	40	11	13.30	12.0		
Smoking (%)	HC	55	18	16.36	—	χ²(2) = 6.00	0.050*
	SCZ	40	11	37.50	—		
IQ	HC	53	20	112.00	11.5	*t*(88) = 4.56	<0.001*
	SCZ	40	11	99.40	16.80		
CPZ (mg/day)	HC	0	73	—	—	—	—
	SCZ	40	11	892.65	854.7		
ACB	HC	0	73	—	—	—	—
	SCZ	40	11	3.40	1.61		
BACS-SC	HC	55	18	54.40	9.30	*t*(90) = 10.46	<0.001*
	SCZ	40	11	35.40	8.81		
FAS	HC	55	18	41.30	6.96	*t*(90) = 2.81	0.006*
	SCZ	40	11	37.48	7.64		
PANSS Positive	HC	0	73	—	—	—	—
	SCZ	40	11	13.95	4.93		
PANSS Negative	HC	0	73	—	—	—	—
	SCZ	40	11	14.43	5.50		
PANSS-Total	HC	0	73	—	—	—	—
	SCZ	40	11	57.30	14.51		

Age, IQ, BACS-SC, and FAS were compared between groups using two-sample t-tests, whereas sex and smoking status were assessed using chi-squared tests. Statistical significance was set at *p* < 0.05, * Statistical significance.

*HC* healthy controls, *SCZ* patients with schizophrenia, *IQ* intelligence quotient, *CPZ* chlorpromazine equivalent dose, *ACB* anticholinergic burden score, *BACS-SC* Brief Assessment of Cognition in Schizophrenia, Symbol Coding Task, *FAS* phonemic verbal fluency task, *PANSS* Positive and Negative Syndrome Scale.

Clinical and cognitive data were available for a subset of participants (55 HC: age range 18–65 years, 40 males; 40 SCZ: age range 19–57 years, 32 males). Participants with and without available cognitive data did not differ significantly in age or sex distribution (*p* > 0.05).

Between-group comparisons revealed no significant differences in age or sex distribution between HC and SCZ participants. However, patients with SCZ showed significantly higher smoking rates, lower IQ scores, and reduced cognitive performance on the BACS-SC and FAS task compared with HC (see Table 1).

In patients with SCZ, the mean age of onset was 20.5 years (*SD* = 8.3), the mean duration of illness was 13.3 years (*SD* = 12.0), the mean CPZ dose was 892.7 mg/day (*SD* = 854.7), and the mean ACB score was 3.4 (*SD* = 1.6).

### Intrinsic functional connectivity of the BFCN

#### Distinct functional connectivity patterns of aBFCN and pBFCN

One-sample *t*-tests revealed distinct intrinsic functional connectivity patterns for the aBFCN and pBFCN in both groups. Across groups, the aBFCN showed connectivity with medial temporal and limbic regions, including the hippocampus and medial prefrontal cortex. In contrast, the pBFCN showed connectivity with regions implicated in salience-related and sensorimotor processing, including the insula, dorsal anterior cingulate cortex, and thalamus.

**Fig. 1:**
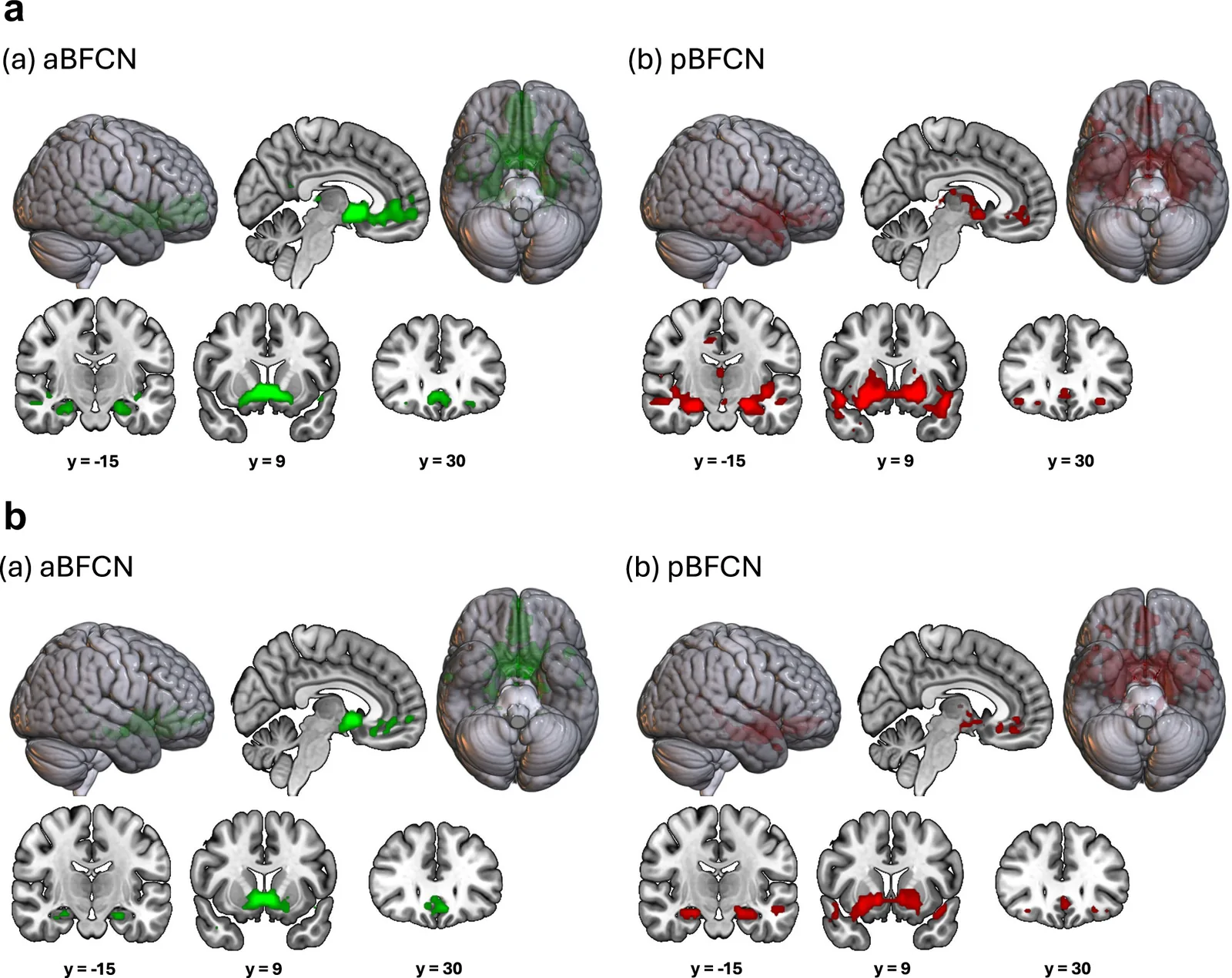
Intrinsic functional connectivity profiles of anterior and posterior basal forebrain cholinergic nuclei in healthy controls and patients with schizophrenia. **a** Voxel-wise intrinsic functional connectivity (iFC) profiles of anterior and posterior basal forebrain cholinergic nuclei (aBFCN; pBFCN) in healthy controls. The aBFCN is shown in green and the pBFCN in red (both *p* < 0.05, FWE-corrected). **b** Corresponding voxel-wise iFC profiles of aBFCN (green) and pBFCN (red) in patients with schizophrenia (both *p* < 0.05, FWE-corrected). In both panels, iFC profiles are illustrated on lateral, medial, and ventral brain surfaces and on representative coronal sections at MNI coordinates *y* = −15, *y* = 9, and *y* = 30.

Voxel-wise iFC profiles for HC and patients with SCZ are shown in Fig. 1. Notably, the iFC patterns identified in HC replicate previous findings in healthy participants^33^.

#### Group differences in pBFCN intrinsic functional connectivity, controlling for age and sex

**Fig. 2:**
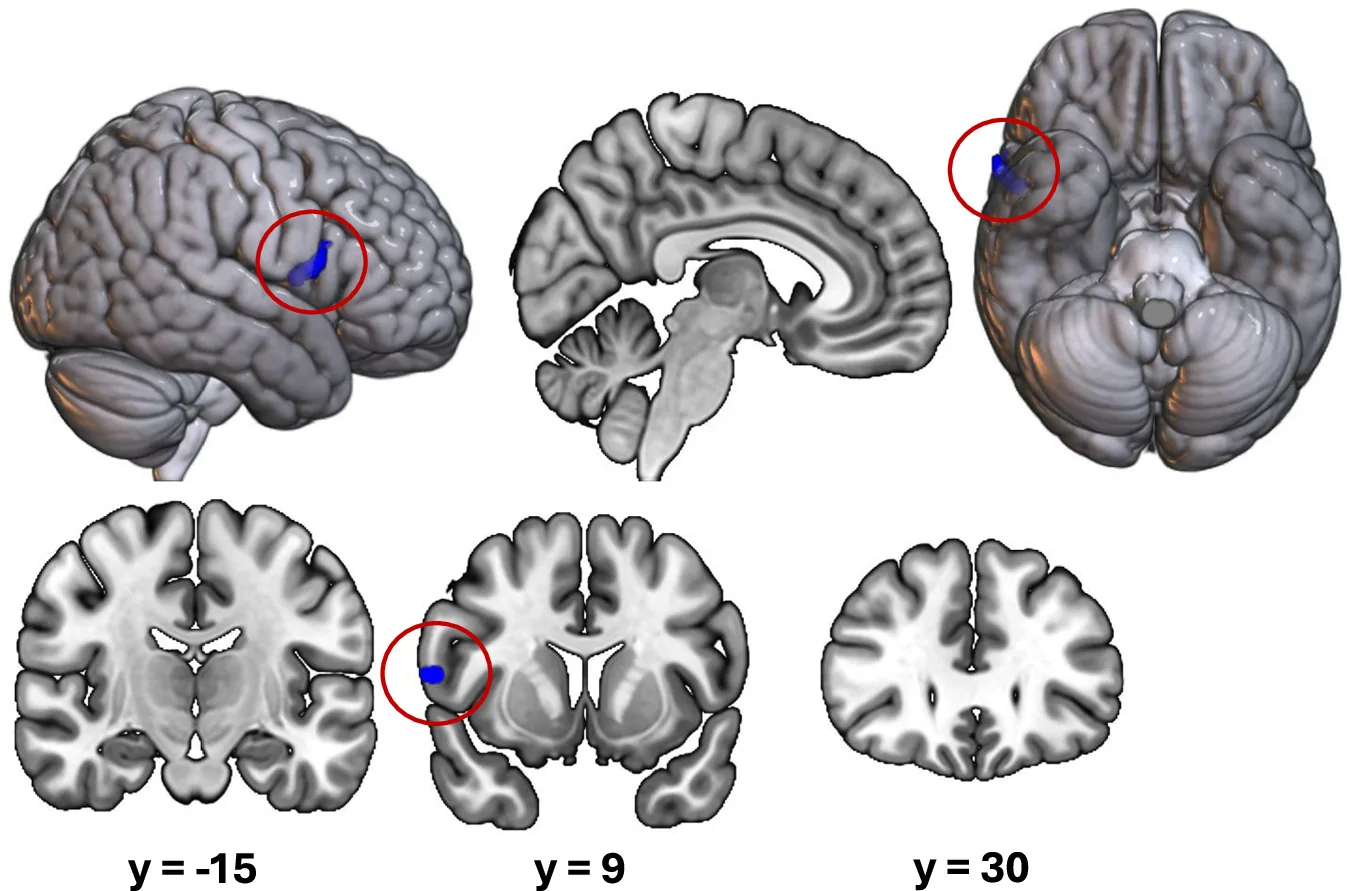
Reduced intrinsic functional connectivity (iFC) of posterior basal forebrain cholinergic nuclei (pBFCN) in schizophrenia. Group differences were assessed using a two-sample *t*-test in SPM12 (voxel-level threshold *p* < 0.001, uncorrected; cluster-level FWE-corrected *p* < 0.05). Patients with schizophrenia showed reduced iFC of the pBFCN in a cluster located in the right inferior frontal gyrus (pars opercularis; BA44 according to the AAL atlas; cluster size = 27 voxels; peak MNI coordinates: *x* = 63, *y* = 15, *z* = 15; peak *t* = 5.10, *p* = 0.018, FWE-corrected).

Voxel-wise group comparisons revealed a significant reduction in pBFCN iFC in patients with SCZ relative to HCs. This effect was localized to a cluster in the right inferior frontal gyrus (pars opercularis; BA44 according to the AAL atlas; cluster size = 27 voxels; peak MNI coordinates: *x* = 63, *y* = 15, *z* = 15; peak *t* = 5.10; *p* = 0.018, FWE-corrected; Fig. 2).

Within this cluster, HC showed slightly positive connectivity (Fisher z-transformed mean ± SD = 0.0215 ± 0.0553), whereas SCZ showed slightly negative connectivity (−0.0407 ± 0.0540), indicating a shift from slightly positive toward inverse pBFCN–IFG connectivity in schizophrenia (Supplementary Fig. 4).

Overlay of the between-group cluster onto the HC pBFCN connectivity map at p < .001 uncorrected showed spatial overlap between the two maps (Supplementary Fig. 3).

#### Group differences in aBFCN intrinsic functional connectivity, controlling for age and sex

No significant group differences were observed for the aBFCN seed.

#### Exploratory molecular mapping of the pBFCN–IFG cluster

**Fig. 3:**
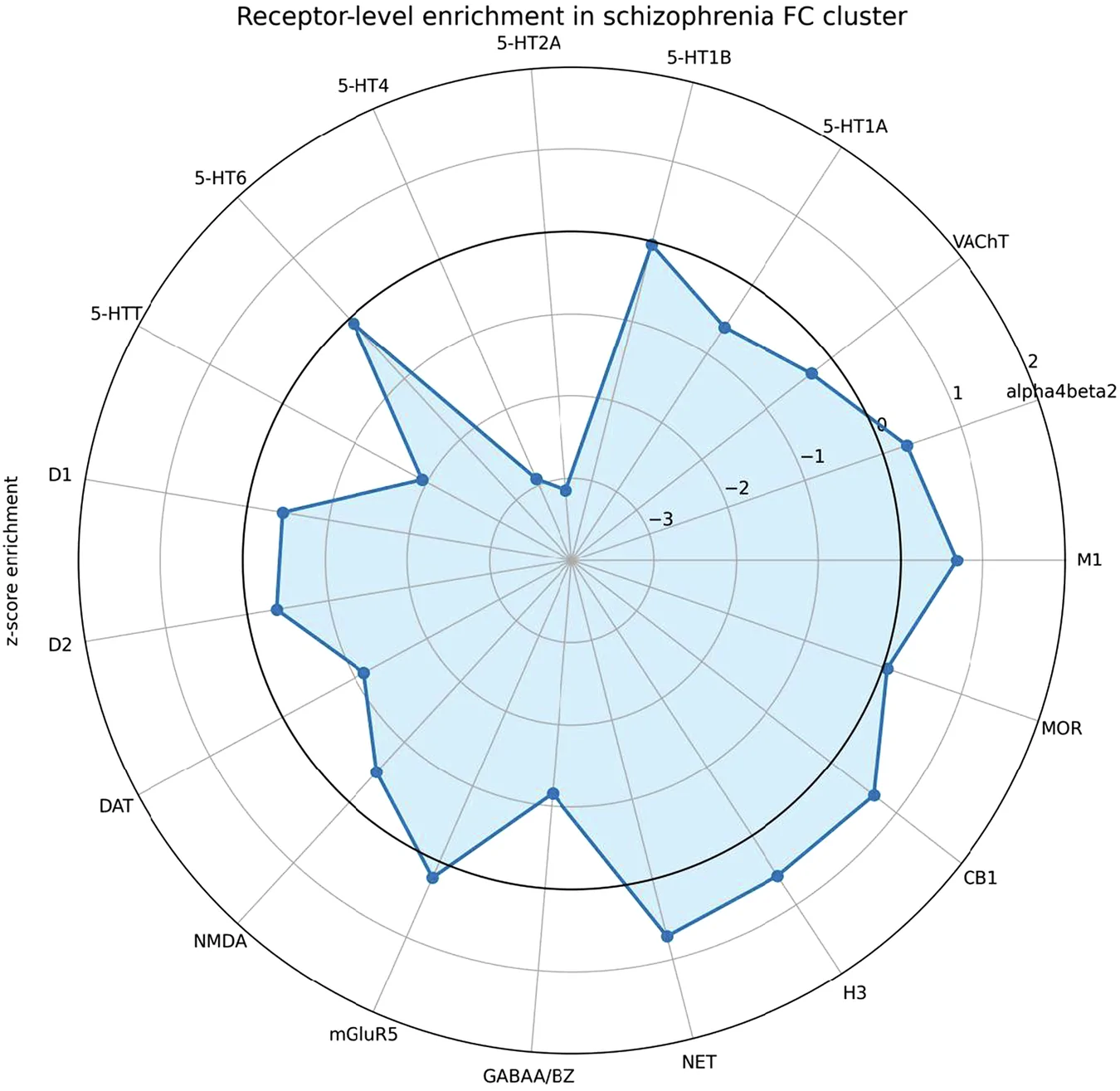
Receptor-level PET enrichment profile of the schizophrenia-related posterior basal forebrain cholinergic nuclei (pBFCN) hypoconnectivity cluster. Spider plot illustrating receptor/transporter enrichment z-scores derived from normative PET neurotransmitter maps of the Hansen atlas^50^. Positive values indicate above-average receptor/transporter density within the schizophrenia-related pBFCN hypoconnectivity cluster relative to the cortical reference distribution. *M1* muscarinic acetylcholine receptor M1, *α4β2* nicotinic acetylcholine receptor α4β2, *VAChT* vesicular acetylcholine transporter, *5-HT1A*, *5-HT1B*, *5-HT2A*, *5-HT4*, *5-HT6* serotonin receptors, *5-HTT* serotonin transporter, *D1* dopamine D1 receptor, *D2* dopamine D2 receptor, *DAT* dopamine transporter, *NMDA* N-methyl-D-aspartate receptor, *mGluR5* metabotropic glutamate receptor 5, *GABAA/BZ* GABAA/benzodiazepine receptor, *H3* histamine H3 receptor, *NET* norepinephrine transporter, *CB1* cannabinoid receptor 1, *MOR* μ-opioid receptor.

Descriptive receptor/transporter mapping showed above-average representation of the cholinergic M1 and α4β2 receptor maps within the pBFCN–IFG cluster, while VAChT was slightly below the cortical average (Fig. 3). Above-average descriptive scores were also observed for several non-cholinergic targets, including NET, H3, CB1, and mGluR5, indicating that the molecular profile was not selectively cholinergic.

In the additional spatial-autocorrelation-preserving null-model analysis, none of the 19 receptor/transporter targets showed significant spatial correspondence with the pBFCN–IFG cluster after FDR correction (all FDR-adjusted *q* > 0.05), and no target reached significance at the uncorrected level (all *p* ≥ 0.168). Specifically, M1 (*p* = 0.208), α4β2 (*p* = 0.331), and VAChT (*p* = 0.846) were not significant. Thus, the descriptive molecular profile was not accompanied by significant preferential spatial correspondence in the spatial-autocorrelation-aware analysis (Supplementary Table 1).

### Associations of BFCN intrinsic functional connectivity with anticholinergic burden and smoking in schizophrenia, controlling for age and sex

Within SCZ, higher ACB was associated with regionally heterogeneous alterations in both BFCN subdivisions. For aBFCN, positive associations involved a large subcortical/ventromedial cluster encompassing accumbens/caudate and subcallosal regions, as well as right superior/middle temporal cortex, posterior cingulate cortex, and right angular gyrus; negative associations involved bilateral frontal opercular/IFG and middle frontal regions, bilateral superior parietal cortex, and right supramarginal cortex. For pBFCN, positive associations involved a large predominantly subcortical cluster encompassing the putamen and adjacent ventral regions, right orbitofrontal cortex, and left inferior temporal/fusiform cortex; negative associations involved right middle frontal and frontal opercular/IFG regions and right superior parietal cortex (all cluster-level FWE-corrected *p* < 0.05). Neither positive nor negative ACB-associated pBFCN clusters spatially overlapped with the primary pBFCN–IFG group-difference cluster (0 overlapping voxels for both contrasts; Supplementary Fig. 2). No significant positive or negative associations with smoking status were observed for either BFCN subdivision.

### Association between pBFCN–IFG connectivity and cognitive performance, controlling for age, sex, IQ, and smoking

**Fig. 4:**
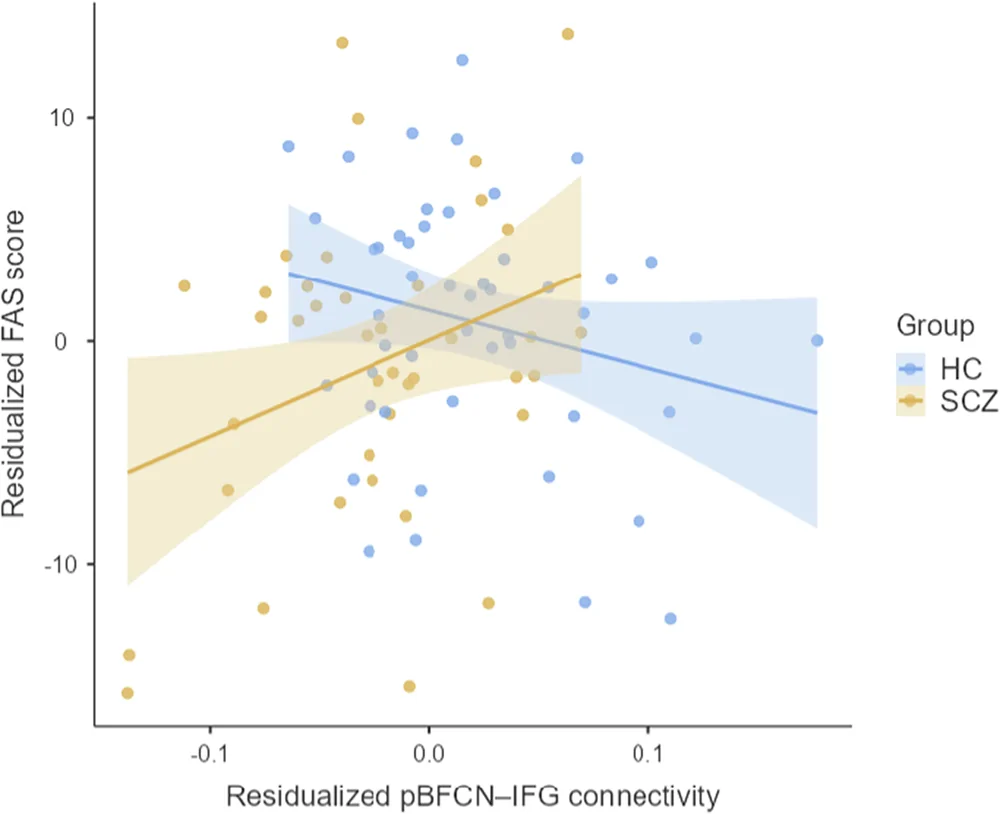
Association between pBFCN–IFG functional connectivity and verbal fluency performance in healthy controls and individuals with schizophrenia. Residualized pBFCN–IFG functional connectivity and FAS scores are shown after adjustment for age, sex, smoking status, and IQ. Lines represent group-specific linear fits for healthy controls (HC) and individuals with schizophrenia (SCZ); shaded areas represent standard errors.

For FAS, the overall model including the Group × pBFCN–IFG interaction was significant, *F*(7, 81) = 4.83, *p* < 0.001, *R*² = 0.295, adjusted *R*² = 0.234 (*N* = 89). Adding the interaction significantly improved model fit (Δ*R*² = 0.054, Δ*F*(1, 81) = 6.25, *p* = 0.014), and this improvement remained significant at the Bonferroni-corrected α = 0.025 (Table 2). Follow-up simple slopes showed a positive association in SCZ (B = 51.48, SE = 23.88, β = 0.327, *t*(34) = 2.16, *p* = 0.038, 95% CI [2.94, 100.02]) and a negative but non-significant association in HC (B = −26.67, SE = 16.52, β = −0.240, *t*(43) = −1.61, *p* = 0.114, 95% CI [−59.99, 6.66]). Partial correlations controlling for the same covariates were *r* = 0.347 (*p* = 0.038) in SCZ and *r* = −0.239 (*p* = 0.114) in HC and differed significantly between groups (Fisher *z* = 2.60, *p* = 0.009; Fig. 4).

**Table 2:** Multiple regression analysis showing a significant interaction between pBFCN–IFG functional connectivity and diagnostic group in predicting verbal fluency (FAS scores).

			95% Confidence Interval				
Predictor	*B*	*SE*	Lower	Upper	*t*	*p*	β
Intercept	19.25	6.07	7.17	31.32	3.17	0.002	—
pBFCN–IFG	−24.42	17.27	−58.78	9.95	−1.41	0.161	−0.21
Group	−1.57	1.71	−4.97	1.84	−0.92	0.363	—
Age	0.02	0.06	−0.09	0.13	0.33	0.744	0.03
Sex	2.56	1.62	−0.66	5.78	1.58	0.117	—
IQ	0.19	0.05	0.09	0.29	3.82	<0.001	0.40
Smoking	3.81	1.63	0.58	7.05	2.35	0.021	—
pBFCN–IFG × Group	68.34	27.34	13.94	122.75	2.50	0.014	—

*N* = 89. The multiple regression model predicting verbal fluency (FAS scores) was significant, *F*(7, 81) = 4.83, *p* < 0.001, *R*² = 0.295, adjusted *R*² = 0.234. Addition of the Group × pBFCN–IFG interaction resulted in Δ*R*² = 0.054, Δ*F*(1, 81) = 6.25, *p* = 0.014. Group was coded 1 = HC and 2 = SCZ; sex was coded 1 = female and 2 = male; smoking status was coded 0 = non-smoker and 1 = smoker. HC, female sex, and non-smoking status served as the respective reference categories. Standardized coefficients are reported for continuous predictors only; standardized coefficients for categorical predictors and factor-related interaction terms are omitted.

*B* unstandardized regression coefficient, *SE* standard error, *CI* confidence interval, *β* standardized regression coefficient, *pBFCN* posterior basal forebrain cholinergic nuclei, *IFG* inferior frontal gyrus, *HC* healthy controls, *SCZ* schizophrenia.

In an additional SCZ sensitivity analysis, further adjustment for PANSS-negative scores, CPZ, and ACB did not significantly improve model fit (Δ*R*² = 0.019, ΔF(3, 31) = 0.29, *p* = 0.835). The magnitude of the positive pBFCN–IFG association with FAS remained largely unattenuated in the fully adjusted model (*B* = 50.54, SE = 25.47, *t*(31) = 1.98, *p* = 0.056).

For BACS-SC, the overall model was significant, *F*(7, 83) = 21.50, *p* < 0.001, *R*² = 0.645, adjusted *R*² = 0.615 (*N* = 91), but the interaction did not improve model fit (Δ*R*² < 0.001, Δ*F*(1, 83) = 0.09, *p* = 0.762; *B* = −10.28, SE = 33.88, *t*(83) = − 0.30, 95% CI [−77.66, 57.11]). For HVLT, the overall model was significant, *F*(7, 82) = 2.99, *p* = 0.008, *R*² = 0.203, adjusted *R*² = 0.135 (*N* = 90), but the interaction was not significant (Δ*R*² = 0.004, Δ*F*(1, 82) = 0.43, *p* = 0.514; *B* = −21.67, SE = 33.09, *t(82)* = −0.65, 95% CI [−87.51, 44.17]). No group-specific follow-up analyses were conducted for BACS-SC or HVLT. Diagnostic checks indicated no substantial violations of model assumptions and no highly influential observations.

### Volume sensitivity analysis

In participants with available pBFCN volume data (*N* = 97; HC: *n* = 56, SCZ: *n* = 41), the group difference in pBFCN–IFG connectivity remained significant after adjustment for TIV-corrected pBFCN volume (*B* = −0.064, SE = 0.011, *t*(92) = −5.59, *p* < 0.001, 95% CI [−0.087, −0.041]). Addition of pBFCN volume did not significantly improve model fit (Δ*R*² = 0.005, Δ*F*(1, 92) = 0.60, *p* = 0.440), and pBFCN volume itself was not significantly associated with pBFCN–IFG connectivity (*B* = 0.018, SE = 0.023, β = .088, *t*(92) = 0.78, *p* = 0.440, 95% CI [−0.028, 0.064]).

## Discussion

The present study identified a selective reduction of pBFCN connectivity with the right IFG in SCZ, whereas no significant aBFCN group difference was detected. The pBFCN–IFG connectivity difference reflected a shift from slightly positive coupling in HC toward inverse coupling in SCZ. Importantly, the association between pBFCN–IFG connectivity and verbal fluency was group-dependent, whereas corresponding interactions were not observed for processing speed or verbal learning and memory. Descriptive molecular mapping showed above-average representation of several cholinergic and non-cholinergic targets, but none showed significant preferential spatial correspondence in the spatial-autocorrelation-aware analysis. Together, these findings demonstrate a selective alteration of pBFCN–IFG connectivity in schizophrenia that is associated with verbal fluency performance.

### Selective pBFCN hypoconnectivity with the right inferior frontal cortex in schizophrenia

The main group difference involved reduced pBFCN connectivity with the right IFG (pars opercularis; BA44), a region implicated in cognitive control and speech/language-related processes^37,38^. The IFG, including the frontoparietal opercular territory encompassing BA44, is an anatomical target of ascending cholinergic projections from the nucleus basalis of Meynert via the lateral cholinergic pathway^52^. Consistent with this established anatomical connectivity, overlay of the between-group cluster onto the HC pBFCN connectivity map at *p* < 0.001 uncorrected showed spatial overlap between the two maps (Supplementary Fig. 3).

The regional specificity of the observed alteration may reflect selective circuit vulnerability within pBFCN–cortical pathways rather than widespread or nonspecific BFCN dysconnectivity. Consistent with this anatomical and functional convergence, the group-dependent cognitive association was observed for phonemic verbal fluency but not for processing speed or verbal learning and memory. This pattern suggests that BFCN-related abnormalities in schizophrenia may preferentially involve specific cortical circuits and their associated cognitive functions rather than producing uniform dysconnectivity across the cortex or generalized associations across cognitive domains.

The present findings should also be considered in the context of previous COBRE studies demonstrating widespread alterations of functional connectivity and network organization in schizophrenia^34–36^. Standard whole-brain parcellations may blur or omit small, anatomically specialized basal forebrain subdivisions. By leveraging a targeted functional parcellation of the aBFCN and pBFCN, the present study complements macro-scale network analyses by examining a specific subcortical–cortical projection system.

### Association with cognitive performance

The association between pBFCN–IFG connectivity and verbal fluency was group-dependent. Higher connectivity was associated with better FAS performance in SCZ, whereas the association was negative but not statistically significant in HC; the group-specific partial correlations differed significantly. Thus, the negative direction in HC should not be interpreted as evidence that stronger pBFCN–IFG connectivity is associated with poorer verbal fluency under physiological conditions. More generally, the present findings do not establish that stronger pBFCN–IFG connectivity supports verbal fluency under normal physiological conditions. Instead, variation in this circuit may become behaviorally relevant in the context of schizophrenia-related circuit dysfunction. This interpretation remains hypothetical because resting-state functional connectivity does not establish the physiological or neurochemical mechanism underlying the group-dependent association.

The Group × connectivity interaction was significant for FAS but not for BACS-SC or, in the additional specificity analysis, HVLT. All connectivity–cognition models adjusted for IQ, reducing the likelihood that the FAS association simply reflects differences in general intellectual ability. However, these findings do not establish strict domain specificity. Cholinergic influences on cognition are not restricted to a single domain, and recent evidence in psychosis demonstrates associations between anticholinergic burden and performance across multiple cognitive domains^53^. Future studies using broader, prospectively defined cognitive batteries or latent measures of general cognitive ability are needed to determine whether BFCN connectivity alterations relate preferentially to specific cognitive processes or broader cognitive impairment.

Importantly, phonemic verbal fluency should not be considered a specific marker of trait-like cholinergic dysfunction. It is a multidetermined measure depending on executive control, lexical retrieval, and processing speed, and may be influenced by clinical state, negative symptoms, and medication-related factors. Additional adjustment for negative symptom severity, antipsychotic dose, and anticholinergic burden left the magnitude of the pBFCN–IFG association with FAS largely unattenuated. Thus, while these factors did not substantially alter the estimated association, the finding should be interpreted cautiously and does not establish a specific or trait-like cholinergic basis for verbal fluency impairment.

### Absence of detectable aBFCN connectivity alterations in schizophrenia

Contrary to our subdivision-specific hypothesis, no significant between-group differences in aBFCN connectivity were identified. This null finding should not be interpreted as evidence for preserved aBFCN function in schizophrenia. More subtle or spatially heterogeneous alterations may not have been detectable at the stringent whole-brain threshold. In addition, medial and inferior temporal regions are particularly susceptible to magnetic-field inhomogeneities and signal dropout in standard BOLD fMRI^54,55^, which may reduce sensitivity to subtle aBFCN connectivity alterations. At the same time, selective pBFCN dysconnectivity may reflect some degree of functional specificity. Previous structural MRI studies have demonstrated alterations involving anterior and posterior BFCN subdivisions^26,27^, but these structural alterations need not translate directly into resting-state functional coupling changes. Larger studies are required to determine whether the absence of detectable aBFCN alterations reflects limited sensitivity or genuine subregional specificity.

### Molecular mapping and neurochemical interpretation

Descriptive molecular mapping showed above-average representation of M1 and α4β2 receptor maps within the pBFCN–IFG cluster. However, above-average descriptive scores were also observed for NET, H3, CB1, and mGluR5, indicating that the profile was not selectively cholinergic. This multimodulatory profile is consistent with the diverse neuromodulatory inputs characteristic of frontal association cortex and should not be interpreted as a distinctive molecular property of the identified IFG region. Furthermore, no molecular target reached statistical significance in the spatial-autocorrelation-aware null analysis, including M1, α4β2, and VAChT. The PET correspondences, therefore, remain descriptive and do not isolate a single neurochemical substrate.

### Implications of basal forebrain circuitry in schizophrenia

The present findings extend previous work on basal forebrain alterations in schizophrenia by demonstrating altered intrinsic functional connectivity between the pBFCN and inferior frontal cortex. As the pBFCN comprises an important source of cortical cholinergic projections, the observed alteration identifies disrupted functional coupling within a cytoarchitectonically defined cholinergic projection system and provides circuit-level evidence of basal forebrain network disruption. However, resting-state functional connectivity reflects statistical dependencies between BOLD signal fluctuations and does not directly quantify acetylcholine release, receptor binding, or cholinergic neurotransmission. Similarly, the normative molecular mapping cannot establish a cholinergic mechanism. Accordingly, the findings establish altered pBFCN-related functional circuitry while its underlying neurochemical mechanisms remain unresolved.

### Replication of subdivision-specific BFCN connectivity

The iFC patterns observed for aBFCN and pBFCN were broadly consistent with previous reports describing distinct functional subdivisions of the basal forebrain^33^. Specifically, the aBFCN showed connectivity with medial temporal and limbic regions, whereas the pBFCN was connected with more lateral cortical regions implicated in salience-related and sensorimotor functions.

These findings support the validity of the two-cluster parcellation of the BFCN and indicate that this subdivision yields reproducible connectivity patterns in an independent dataset. By applying this framework in a clinical sample, the present study extends prior work by suggesting that this organizational scheme is robust enough to investigate disorder-related alterations.

### Strengths and limitations

A major strength is the use of an established functional parcellation of the BFCN, allowing separate investigation of anterior and posterior subdivisions. The aBFCN and pBFCN showed clearly differentiated whole-brain connectivity profiles that qualitatively reproduced the functional dissociation reported previously^33^. Nevertheless, given the small anatomical size and high surface-to-volume ratio of the BFCN, partial-volume effects and contributions from adjacent structures cannot be completely excluded. Preprocessing included aCompCor-based removal of white-matter and cerebrospinal-fluid nuisance signals, and the differentiated connectivity profiles argue against the results being predominantly driven by nonspecific signal bleed, although partial-volume effects cannot be fully excluded at the applied resolution. Future submillimeter high-field fMRI may further reduce these effects.

Seed-based resting-state functional connectivity reflects statistical correlations between regional signal fluctuations and does not establish direct or causal interactions. Thus, the pBFCN–IFG association may also be influenced by shared inputs or fluctuations arising from other interconnected cortical, subcortical, or neuromodulatory regions. Moreover, the pBFCN–IFG alteration should be considered within the broader distributed dysconnectivity of schizophrenia rather than as an abnormality unique to BFCN-related circuitry; complementary alternative-seed analyses were not performed, so specificity to BFCN connectivity cannot be established.

Patients had relatively long illness duration and comparatively high antipsychotic exposure. Whole-brain analyses showed that ACB was associated with substantial, regionally heterogeneous alterations of both aBFCN and pBFCN connectivity. Importantly, ACB-associated pBFCN clusters were spatially dissociable from the primary pBFCN–IFG group difference, reducing concern that the primary finding simply reflects anticholinergic burden. No significant smoking associations were detected, although binary self-reported smoking status may provide lower sensitivity and variance than the continuous ACB measure. Potential effects of cumulative medication exposure and illness progression, nevertheless, cannot be fully excluded.

The pBFCN–IFG difference also remained significant after adjustment for individual pBFCN volume, suggesting that it was not statistically accounted for by individual variation in pBFCN volume. Structural and resting-state functional measures capture different aspects of brain organization and may provide complementary information, although their mechanistic relationship cannot be determined from cross-sectional data.

A further limitation concerns the molecular mapping. Normative PET atlases were derived from independent healthy samples rather than participant-specific molecular imaging. The pBFCN–IFG cluster was relatively small (27 voxels on the 3-mm grid), and receptor-specific spatial-null analyses included only the 11–20 cluster voxels falling within each valid PET domain. This limited and target-dependent coverage may reduce sensitivity and comparability. The molecular findings should therefore be considered exploratory despite control for spatial autocorrelation. Finally, motion-related exclusions and the reduced cognitive subsample may limit generalizability.

### Future directions

Future studies should replicate these findings in larger and more clinically diverse samples, including first-episode and minimally medicated patients. Task-based and high-field fMRI may help clarify the functional role and anatomical specificity of BFCN-related circuitry. Participant-specific molecular PET combined with functional connectivity will be important for determining whether individual differences in pBFCN-related circuitry are associated with cholinergic receptor availability or neurotransmission and for resolving the neurochemical drivers of the observed connectivity alterations.

## Conclusion

In summary, the present study identifies altered functional connectivity between the pBFCN and right inferior frontal cortex in schizophrenia. The group-dependent association with verbal fluency suggests that variation in pBFCN–IFG connectivity may be behaviorally relevant in schizophrenia. Exploratory molecular mapping showed descriptive correspondence with several normative receptor/transporter distributions, including cholinergic targets; however, none showed significant preferential spatial correspondence after accounting for spatial autocorrelation and multiple comparisons. These findings establish altered pBFCN-related functional circuitry in schizophrenia and highlight the need for participant-specific molecular imaging to resolve its precise neurochemical drivers.

## Supplementary information

Supplementary Material

## Data Availability

The data analyzed in this study were obtained from the publicly available Center for Biomedical Research Excellence (COBRE) dataset. The COBRE dataset is available through the COINS Data Exchange (https://coins.trendscenter.org) and the SchizConnect platform (http://schizconnect.org), subject to the respective data access policies.

## Code availability

Custom code used for the analyses reported in this study is available from the corresponding author upon reasonable request.
