# Supplementary material for: Posterior-lateral basal forebrain cholinergic nuclei hypoconnectivity in the inferior frontal cortex is associated with verbal fluency deficits in schizophrenia
Source: Schizophrenia (Heidelb). 2026 Nov 11;12(1):89. doi: 10.1038/s41537-026-00824-0 (PMC13754921; doi:10.1038/s41537-026-00824-0)
Supplement: Supplementary file 1 [file 41537_2026_824_MOESM1_ESM.docx]

**Posterior-lateral basal forebrain cholinergic nuclei hypoconnectivity in the inferior frontal cortex is associated with verbal fluency deficits in schizophrenia**

Sofia Eickhoff^1^, Alexandra Korda^1^, Christina Andreou^1^, Stefan Borgwardt^1 *^, Mihai Avram^1 *^

^1^Department of Psychiatry and Psychotherapy, University of Luebeck, Luebeck, Germany

* Equal contribution

Corresponding Author:

Mihai Avram, Ph.D.

Department of Psychiatry and Psychotherapy, University of Luebeck, Luebeck, Germany

*E-Mail:* [mihai.avram@uksh.de](mailto:mihai.avram@uksh.de) | Tel: +49 451 500-98715

**Supplementary Material**

**Supplementary Methods**
***MRI acquisition parameters***

Structural and functional MRI data were acquired on a 3T Siemens Trio scanner using a 12-channel radiofrequency coil. High-resolution T2*-weighted functional images were obtained with a gradient-echo echo-planar imaging sequence (TE = 29 ms, TR = 2 s, flip angle = 75°, slice thickness = 3.5 mm, slice gap = 1.05 mm, field of view = 240 mm, matrix size = 64 × 64, voxel size = 3.75 × 3.75 × 4.55 mm³). Detailed acquisition parameters are available in the COBRE dataset documentation (37). The resting-state scan comprised 149 volumes. During scanning, participants were instructed to keep their eyes open and fixate on a central cross.

**Supplementary Figures**

**Figure S1.** Basal forebrain cholinergic nuclei (BFCN) parcellation for a two-cluster solution.
Coronal slices from anterior to posterior showing the anterior–medial BFCN cluster (aBFCN, green) and posterior–lateral BFCN cluster (pBFCN, red). Slice positions are indicated by Montreal Neurological Institute (MNI) y-coordinates.

**Figure S2.**  Whole-brain associations of anticholinergic burden with aBFCN and pBFCN functional connectivity and their spatial relationship to the primary pBFCN group-difference cluster.

Whole-brain clusters showing significant positive and negative associations between ACB and aBFCN and pBFCN connectivity within the SCZ group are displayed alongside the pBFCN–IFG cluster showing reduced connectivity in SCZ compared with HC. The primary pBFCN group-difference cluster is shown in blue, positive associations with ACB in green, and negative associations with ACB in red. ACB-associated clusters were identified using a voxel-level threshold of p < .001 uncorrected, with a minimum cluster extent of k ≥ 15 voxels and cluster-level FWE correction at p < .05. Neither the positive nor negative ACB-associated pBFCN clusters spatially overlapped with the primary pBFCN group-difference cluster.

**Figure S3.** Spatial relationship between pBFCN functional connectivity in healthy controls and the primary pBFCN group-difference cluster.

The whole-brain pBFCN functional connectivity map in HC is displayed at a voxel-level threshold of p < .001 uncorrected, alongside the pBFCN–IFG cluster showing reduced connectivity in SCZ compared with HC. The whole-brain pBFCN connectivity map in HC is shown in red, and the primary pBFCN group-difference cluster is shown in blue. The overlay illustrates the spatial relationship between the schizophrenia-related pBFCN–IFG connectivity alteration and the physiological pBFCN connectivity pattern observed in HC.

**Figure S4.** Distribution of pBFCN–IFG functional connectivity in healthy controls and patients with schizophrenia.

Boxplots show Fisher z-transformed pBFCN–IFG functional connectivity estimates in healthy controls (HC; n = 73) and patients with schizophrenia (SCZ; n = 51). The pBFCN–IFG cluster was identified in the between-group analysis controlling for age and sex. Mean ± SD connectivity was 0.0215 ± 0.0553 in HC and −0.0407 ± 0.0540 in SCZ. FC = functional connectivity; pBFCN = posterior basal forebrain cholinergic nuclei; IFG = inferior frontal gyrus.

**Supplementary Table 1**

*Spatial Autocorrelation-Preserving Null-Model Results for Molecular Targets Within the pBFCN–IFG* Cluster

| Target | Statistic | *p* | FDR *q* | Voxels (of 27) |
| --- | --- | --- | --- | --- |
| M1 | 0.88 | .208 | .95 | 20 |
| α4β2 | 0.85 | .331 | .95 | 20 |
| VAChT | 0.16 | .846 | .95 | 20 |
| 5-HT1A | −0.18 | .802 | .95 | 20 |
| 5-HT1B | 0.47 | .551 | .95 | 20 |
| 5-HT2A | 0.81 | .399 | .95 | 11 |
| 5-HT4 | 0.39 | .715 | .95 | 11 |
| 5-HT6 | 0.37 | .591 | .95 | 20 |
| 5-HTT | −0.43 | .619 | .95 | 11 |
| D1 | −0.12 | .834 | .95 | 20 |
| D2 | −0.05 | .990 | .99 | 20 |
| DAT | −0.20 | .846 | .95 | 20 |
| NMDA | −0.14 | .898 | .95 | 20 |
| mGluR5 | 0.69 | .379 | .95 | 20 |
| GABAA/BZ | −0.20 | .822 | .95 | 20 |
| NET | 1.00 | .168 | .95 | 20 |
| H3 | 0.69 | .335 | .95 | 20 |
| CB1 | 0.79 | .299 | .95 | 20 |
| MOR | 0.50 | .475 | .95 | 20 |

*Note.* Statistic = mean standardized molecular-map value within voxels shared by the pBFCN–IFG cluster and the respective receptor-specific valid cortical PET domain. For each target, 500 spatial surrogate maps were generated using BrainSMASH to approximately preserve the spatial autocorrelation structure of the empirical molecular distribution. Two-sided empirical *p* values were derived from the surrogate distributions, and *q* values were obtained using Benjamini–Hochberg false discovery rate (FDR) correction across the 19 molecular targets. No target was significant after FDR correction. pBFCN = posterior basal forebrain cholinergic nuclei; IFG = inferior frontal gyrus; PET = positron emission tomography; M1 = muscarinic acetylcholine receptor M1; α4β2 = α4β2 nicotinic acetylcholine receptor; VAChT = vesicular acetylcholine transporter; 5-HT = serotonin; 5-HTT = serotonin transporter; D1 = dopamine D1 receptor; D2 = dopamine D2 receptor; DAT = dopamine transporter; NMDA = N-methyl-D-aspartate receptor; mGluR5 = metabotropic glutamate receptor 5; GABAA/BZ = γ-aminobutyric acid type A/benzodiazepine receptor; NET = norepinephrine transporter; H3 = histamine H3 receptor; CB1 = cannabinoid receptor 1; MOR = μ-opioid receptor.
